# Inhibition of the RAF/MEK/ERK Signaling Cascade in Pancreatic Cancer: Recent Advances and Future Perspectives

**DOI:** 10.3390/ijms25031631

**Published:** 2024-01-28

**Authors:** Christos Adamopoulos, Donatella Delle Cave, Athanasios G. Papavassiliou

**Affiliations:** 1 Department of Biological Chemistry, Medical School, National and Kapodistrian University of Athens, 11527 Athens, Greece; cadamop@med.uoa.gr; 2 Department of Oncological Sciences, Icahn School of Medicine at Mount Sinai, New York, NY 10029, USA; 3 Institute of Genetics and Biophysics ‘Adriano Buzzati-Traverso’, CNR, 80131 Naples, Italy

**Keywords:** pancreatic cancer, RAF/MEK/ERK pathway, small-molecule inhibitors, KRAS, targeted therapy

## Abstract

Pancreatic cancer represents a formidable challenge in oncology, primarily due to its aggressive nature and limited therapeutic options. The prognosis of patients with pancreatic ductal adenocarcinoma (PDAC), the main form of pancreatic cancer, remains disappointingly poor with a 5-year overall survival of only 5%. Almost 95% of PDAC patients harbor Kirsten rat sarcoma virus (KRAS) oncogenic mutations. KRAS activates downstream intracellular pathways, most notably the rapidly accelerated fibrosarcoma (RAF)/mitogen-activated protein kinase kinase (MEK)/extracellular signal-regulated kinase (ERK) signaling axis. Dysregulation of the RAF/MEK/ERK pathway is a crucial feature of pancreatic cancer and therefore its main components, RAF, MEK and ERK kinases, have been targeted pharmacologically, largely by small-molecule inhibitors. The recent advances in the development of inhibitors not only directly targeting the RAF/MEK/ERK pathway but also indirectly through inhibition of its regulators, such as Src homology-containing protein tyrosine phosphatase 2 (SHP2) and Son of sevenless homolog 1 (SOS1), provide new therapeutic opportunities. Moreover, the discovery of allele-specific small-molecule inhibitors against mutant KRAS variants has brought excitement for successful innovations in the battle against pancreatic cancer. Herein, we review the recent advances in targeted therapy and combinatorial strategies with focus on the current preclinical and clinical approaches, providing critical insight, underscoring the potential of these efforts and supporting their promise to improve the lives of patients with PDAC.

## 1. Introduction

**Figure 1:**
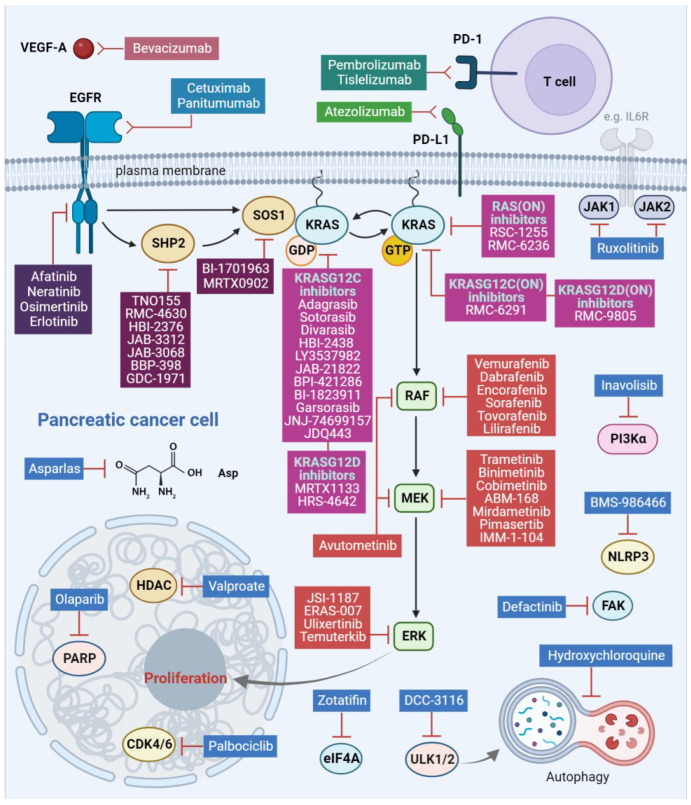
RAF/MEK/ERK pathway inhibitors and combination therapies under clinical evaluation for pancreatic cancer. EGFR, epidermal growth factor receptor; SHP2, Src homology 2 domain-containing phosphatase 2; SOS1, Son of sevenless homolog 1; KRAS, Kirsten rat sarcoma viral oncogene homolog; GDP, guanosine diphosphate; GTP, guanosine triphosphate; RAF, rapidly accelerated fibrosarcoma; MEK, mitogen-activated protein kinase kinase; ERK, extracellular signal-regulated kinase; CDK4/6, cyclin-dependent kinase 4/6; HDAC, histone deacetylase; PARP, poly-adenosine diphosphate (ADP) ribose polymerase; eIF4A, eukaryotic translation initiation factor 4A; ULK1/2, unc-51-like autophagy-activating kinases 1 and 2; NLRP3, Nod-like receptor protein 3; FAK, focal adhesion kinase; JAK1/2, Janus kinase 1/2; PD-1, programmed cell death protein 1; PD-L1, programmed death ligand 1; VEGF-A, vascular endothelial growth factor A; PI3Kα, phosphoinositide 3-kinase α; IL6R, interleukin 6 receptor; Asp, asparagine. This figure was created using the tools provided by BioRender.com (accessed on 21 January 2024).

Pancreatic cancer is one of the deadliest tumors and is expected to become the second leading cause of cancer-related mortality in the US. The 5-year overall survival (OS) of patients with pancreatic ductal adenocarcinoma (PDAC), the most common form of pancreatic cancer, has only minimally improved to only 11%, presenting a modest improvement compared to other malignancies [1,2]. The role of the rapidly accelerated fibrosarcoma (RAF)/mitogen-activated protein kinase kinase (MEK)/extracellular signal-regulated kinase (ERK) pathway as the main RAS effector pathway in initiation and progression of pancreatic cancer is well established [3]. Targeted therapy using small-molecule inhibitors against components of the RAF/MEK/ERK pathway has shown significant potential for PDAC. The Kirsten rat sarcoma virus (KRAS) mutation is a hallmark of PDAC, and only recently has there been progress in drug development, with compounds that directly target the once considered “undruggable” RAS. These compounds include KRAS-mutant-specific inhibitors, that are foreseen to change the landscape in PDAC management [4] (Figure 1).

Here, we discuss rational treatment approaches with the currently available therapeutic options for PDAC patients, including novel targeting strategies using current and new compounds. We focus on the combinatorial strategies and the current clinical attempts for evaluation of the RAF/MEK/ERK pathway inhibitors that are currently in clinical development. This is significant because there is an urgent need to establish new frameworks and improve future treatments. Our aim is to contribute to understanding the complexity of the RAF/MEK/ERK pathway inhibition, which holds substantial promise for developing effective treatment modalities against this aggressive malignancy.

## 2. RAF/MEK/ERK Signaling Pathway in Pancreatic Cancer

The RAF/MEK/ERK pathway which controls cell growth, differentiation and survival is often upregulated in pancreatic cancer. The orchestrator of this upregulation is the small GTPase KRAS, which is mutated in 95% of patients with pancreatic cancer [2]. The most common KRAS mutations in PDAC are substitutions in position G12, with KRASG12D (41%), KRASG12V (34%) and KRASG12R (16%) being the most frequent and G12C (1–2%) the least [5]. KRAS in its active GTP-bound form promotes RAF kinase activation through dimerization and phosphorylation, resulting in phosphorylation of its substrate MEK kinase. MEK phosphorylates and activates the terminal kinase ERK. Activated ERK regulates growth-promoting transcription [2]. The RAF/MEK/ERK pathway is the key effector pathway for initiation and progression of KRAS-driven PDAC [3]. Therefore, apart from targeted efforts against the key members of the RAF/MEK/ERK pathway, several drugs, targeting different components of this pathway, including the upstream epidermal growth factor receptor (EGFR) family members and the RAF/MEK/ERK pathway regulators Src homology-containing protein tyrosine phosphatase 2 (SHP2) and Son of sevenless homolog 1 (SOS1), have been explored extensively for therapeutic intervention in PDAC [2,3,4,5] (Figure 1).

## 3. Targeting Strategies

### 3.1. EGFR Family Inhibition

Initial efforts were directed against the upstream frequently dysregulated EGFR/human epidermal growth factor receptor 2 (HER2 or ERBB2) signaling. Erlotinib, an EGFR inhibitor, combined with gemcitabine, a first-line chemotherapy, in patients with advanced PDAC, showed modest survival benefits [6]. When erlotinib was combined with gemcitabine together with nab-paclitaxel, a tubulin-polymerization stabilizer, it exhibited some clinical activity despite the observed toxicities [7]. However, combining cetuximab, a monoclonal antibody against EGFR, with gemcitabine did not show improved outcome [8]. Similarly, the combination of lapatinib, a dual tyrosine kinase inhibitor against both HER2 and EGFR, with gemcitabine or capecitabine did not demonstrate any efficacy [9,10]. To improve these results, a second-generation ERBB family inhibitor, afatinib, was used in combination with gemcitabine. Afatinib binds covalently to cysteine 797 of the EGFR and the corresponding cysteines 805 and 803 in HER2 and human epidermal growth factor receptor 4 (ErB4/HER4), respectively, inhibiting downstream signaling from all homo- and heterodimers formed by ERBB family members. However, again, this combination did not show any efficacy [11]. Subsequent clinical studies, based on preclinical synergistic evidence, assessed the EGFR inhibition in combination with components of the RAF/MEK/ERK pathway such as BRAF and MEK. These studies combined erlotinib with either sorafenib [12], a multikinase RAF inhibitor, or selumetinib [13], a MEK inhibitor, but showed modest activity. More recently, the addition of panitumumab, an EGFR monoclonal antibody, to erlotinib and gemcitabine demonstrated a small but significantly prolonged overall survival, despite the observed toxicities [14]. Overall, these studies did not show sufficient evidence of effectiveness. This agrees with the conclusions from a retrospective analysis showing that EGFR and KRAS alterations were not predictive for patient benefit from anti-EGFR therapy [15]. However, in a preclinical study, the highly selective irreversible EGFR/HER2 inhibitor neratinib suppressed KRAS mutant levels in PDAC cells [16]. The efficacy of neratinib in combination with valproate, a histone deacetylase (HDAC) inhibitor, is being evaluated in a clinical trial in patients with advanced RAS-mutated solid tumors (Table 1).

### 3.2. RAF/MEK/ERK Pathway Component Inhibition

#### 3.2.1. RAF Inhibition

Early attempts to target BRAF in unselected patients with advanced PDAC, using the BRAF inhibitor sorafenib in combination with gemcitabine did not show any benefit [17]. This is possibly explained by the fact that sorafenib is a multikinase inhibitor and its clinical activity is generally attributed to off-target inhibition. The use of the current clinically available BRAF inhibitors (vemurafenib, dabrafenib and encorafenib) is FDA approved for BRAFV600E-mutant metastatic melanoma but not for RAS-mutant tumors. BRAFV600E oncoprotein signals as a monomer and current BRAF inhibitors target and inhibit BRAF monomers. However, this selectivity limits their effectiveness in RAS-driven tumors, where RAFs (BRAF and CRAF) signal as dimers [18]. Additionally, in RAS-mutant tumors these RAF inhibitors promote paradoxical activation of the mitogen-activated protein kinase (MAPK) signaling by inducing wild-type RAF dimerization [19]. Next-generation RAF inhibitors that inhibit both dimers and monomers are currently in clinical development. These RAF inhibitors induce minimal paradoxical activation and show preclinical activity in RAS-mutant tumors [18,20,21,22]. A clinical trial testing the efficacy of the next-generation RAF inhibitor lilirafenib, including KRAS-mutant PDAC patients, reported stable disease as best response [23]. Additionally, a second current clinical trial is assessing the combination of lilirafenib with the MEK inhibitor mirdametinib in patients with advanced or refractory solid tumors (Table 1). Another clinical trial is evaluating combined vemurafenib and sorafenib treatment in individuals with KRAS-mutant PDAC who have progressed on standard chemotherapy (Table 1).

Activating BRAF alterations make up approximately 30% of KRAS wild-type PDAC and 2% of all PDAC cases [24]. These most commonly include substitutions in position V600, most commonly BRAFV600E. BRAF mutations are mutually exclusive with KRAS mutations and are typically associated with poor prognosis [25]. Multiple preclinical BRAF-mutated models suggest that these alterations can be targeted with combination of BRAF and MEK inhibitors [20,26,27]. Furthermore, BRAFV600E expression in a genetically engineered mouse model of PDAC was sufficient to induce the formation of pancreatic intraepithelial neoplasia lesions, revealing the central role of the RAF/MEK/ERK pathway in PDAC tumorigenesis [3]. Additionally, in a patient-derived orthotopic mouse model of PDAC, treatment with the MEK inhibitors trametinib or cobimetinib resulted in tumor suppression [28]. Molecular targeting of BRAFV600E in KRAS wild-type PDAC, using BRAF and MEK inhibitor, has been reported in several case reports in which patients progressed after first lines of chemotherapy. A case report with a patient with BRAF-mutant advanced PDAC reported objective tumor response to combined vemurafenib plus trametinib treatment [29]. Li et al. reported a case of a patient with metastatic BRAFV600E-mutant PDAC who achieved almost a complete response to dabrafenib plus trametinib treatment. Notably, the patient was rechallenged successfully with the regimen after relapse [30]. Two BRAF-mutant PDAC patients showed a significant reduction in carbohydrate antigen 19-9 levels, a PDAC-associated tumor antigen, following co-treatment with dabrafenib plus trametinib [31]. Wang et al. reported a partial response in the case of advanced metastatic PDAC, after vemurafenib plus trametinib administration [32]. In a recent case report, two patients with BRAFV600E-mutant PDAC exhibited a favorable response to dabrafenib and trametinib co-treatment [33]. Ardalan et al. reported that the addition of the MEK inhibitor cobimetinib to gemcitabine and nab-paclitaxel in BRAF-mutant patients was followed by a complete response to therapy for 16 months [34]. Furthermore, a clinical trial is underway evaluating the combination of encorafenib with the MEK inhibitor binimetinib in BRAFV600E-mutant PDAC patients (Table 1).

#### 3.2.2. MEK Inhibition

Despite the promising preclinical evidence suggesting potent MAPK pathway inhibition, using MEK inhibitors in PDAC, early clinical trials showed limited efficacy. MEK inhibitors, trametinib or pimasertib, in combination with gemcitabine did not show any benefit when compared with gemcitabine alone [35,36]. Of note, the combination of the MEK inhibitor refametinib with gemcitabine was well tolerated and resulted in an objective response rate of 23%, with improved outcomes for KRAS wild-type patients [37]. In contrast, the assessment of selumetinib versus chemotherapy with capecitabine or the dual MEK and protein kinase B (AKT) kinase inhibition with selumetinib and the AKT inhibitor MK-2206 versus oxaliplatin-5-flourouracil-based chemotherapy in patients with advanced PDAC did not show any efficacy [38,39]. Another study on the combination of trametinib with the mammalian target of rapamycin (mTOR) inhibitor everolimus showed modest clinical efficacy, although it was unable to define optimal doses for the two compounds [40]. Moreover, when trametinib was combined with the CDK4/6 inhibitor ribociclib there was no benefit, and the study was terminated [41]. Likewise, in a combination of binimetinib with either the poly-adenosine diphosphate (ADP) ribose polymerase (PARP) inhibitor talazoparib or the programmed death 1 (PD-1) ligand 1 (PD-L1) inhibitor avelumab in patients with metastatic PDAC, no objective responses were observed [42]. Interestingly, preclinical evidence suggests that pancreatic tumors with KRASG12R, the third most common KRAS mutation in PDAC (16%), are more sensitive to MEK or ERK inhibition [43]. This is supported by the documented clinical benefit for patients with KRASG12R-mutant PDAC treated with MEK inhibitors [44,45]. More recently, a phase I clinical trial evaluated ABM-168, a novel small-molecule, allosteric, highly selective MEK inhibitor in adults with advanced solid tumors, including pancreatic carcinoma, who had confirmed RAS, RAF or neurofibromatosis type 1 (NF-1) mutations (Table 1). Another ongoing clinical study is testing the MEK inhibitor cobimetinib in combination with the enzyme calaspargase pegol-mnkl (asparlas) that blocks the biosynthesis of the non-essential amino acid asparagine, leading to starvation of cancer cells (Table 1) [46].

#### 3.2.3. ERK Inhibition

The clinical development of ERK inhibitors raised the hope that direct ERK inhibition could block the MAPK pathway oncogenic transcriptional output. However, early clinical trials using ERK inhibitors against RAS-mutant tumors, including PDAC, were unsuccessful [47,48]. In a recent study, ERK inhibition induced autophagy in KRAS-mutant PDAC and the dual ERK and autophagy inhibition, using SCH772984 and hydrochloroquine, respectively, resulted in enhanced anti-tumor activity in PDAC preclinical models [43,49]. Several clinical studies are testing the synergistic effect of combining hydroxychloroquine with binimetinb, trametinib or the ERK inhibitor temuterkib (Table 1). Cyclin-dependent kinase 4/6 (CDK4/6) is a downstream target of activated/phosphorylated ERK and there is evidence for anti-tumor activity of the dual ERK and CDK4/6 inhibition, using ulixertinib and palbociclib, respectively (Table 1) [50,51] (Figure 1).

### 3.3. RAF/MEK/ERK Pathway Regulator Inhibition

#### 3.3.1. SHP2 Inhibition

The discovery of SHP2 inhibitors revealed the dependency of KRAS-mutant tumors in SHP2 [52]. In addition, several studies demonstrated that SHP2 inhibition prevents the receptor tyrosine kinase (RTK)-mediated development of adaptive resistance caused by MEK or BRAF inhibitors [53,54]. Accordingly, co-targeting SHP2 and MEK or ERK using small-molecule inhibitors has been investigated preclinically, showing promising results in various KRAS-mutant tumors, including PDAC [52,53,54,55,56]. In another strategy, SHP2 inhibitors are combined with the recently developed allele-specific KRASG12C inhibitors to overcome the development of adaptive resistance mediated by wild-type RAS [57]. This approach has been evaluated by two clinical trials testing the combination of a SHP2 inhibitor with a KRASG12C inhibitor, TNO155 with MRTX849 and JAB-3312 with JAB-21822, respectively, in KRASG12C-mutant patients with advanced solid tumors (Table 1). Another aspect of SHP2 inhibition is its reported immunomodulatory function [58]. Based on this evidence, the combination of SHP2 and KRASG12C inhibitors can promote anti-tumor immunity by disrupting MAPK-activating signals from the tumor microenvironment to cancer cells [59].

#### 3.3.2. SOS1 Inhibition

In preclinical PDAC models, it has been shown that SOS1 is essential for the survival of RAS-mutated cancer cells [60]. Small-molecule SOS1 inhibitors that disrupt the SOS1–RAS interaction have been under development for the treatment of KRAS-mutated cancers. Recently, a selective SOS1 inhibitor, BI-3406, has been reported to reduce GTP-bound RAS levels and tumor growth across KRAS-driven cancer models [61,62]. Moreover, the combined treatment of BI-3406 with trametinib resulted in sustained RAF/MEK/ERK pathway inhibition and suppression of tumors in KRAS-mutated xenograft models, overcoming pathway feedback reactivation [62]. The corresponding clinical compound BI-1701963 was tested in a clinical trial for KRAS-mutated solid tumors, including PDAC, with preliminary data demonstrating good tolerability and modest activity [63]. The current second phase of the study is evaluating the effectiveness of the combination of BI-1701963 with trametinib (Table 1).

### 3.4. KRAS Inhibition

Sotorasib is a first-in-class small-molecule inhibitor developed to selectively target KRASG12C, providing evidence for in vivo activity [64]. Adagrasib, another KRASG12C inhibitor, has shown clinical activity in KRASG12C-mutated tumors, including PDAC [65]. Both inhibitors, FDA-approved for *KRAS*G12C-mutant non-small cell lung cancer (NSCLC), trap *KRAS*G12C in its inactive GDP-bound state and are now listed in the national comprehensive cancer network (NCCN) clinical practice guidelines as for additional *KRASG12C-*mutant histologies, including pancreatic and colorectal cancers [66]. Another more potent GDP-bound *KRASG12C* inhibitor, divarasib, in combination with various anti-cancer therapies (Table 1), has shown promising clinical benefit in a small cohort of patients with pancreatic adenocarcinoma harboring the KRASG12C mutation [67]. However, the low prevalence of KRASG12C mutation in PDAC (1–2%) limits the applicability of this approach. Luckily, MRTX1133, a “game-changer” compound, has been developed selectively targeting KRASG12D [68]. MRTX1133 is currently under clinical evaluation, while other novel compounds targeting KRASG12D as well (HRS-4642, RMC-9805) are being assessed in phase I clinical trials (Table 1). A novel non-covalent pan-KRAS inhibitor prevents the activation of wild-type KRAS and a range of KRAS mutants, excluding G12R and Q61L/K/R while sparing NRAS and HRAS isoforms (Kim). This pan-KRAS inhibitor showed preclinical anti-tumor activity in various models, indicating broad therapeutic implications in patients with KRAS-driven cancers, including pancreatic cancer [69]. ADT-007, another pan-KRAS inhibitor, that inhibits GTP binding to both mutated and wild-type KRAS, blocks oncogenic KRAS signaling and modulates T cell activation in preclinical PDAC in vitro and in vivo models [70]. Recently, tricomplex inhibitors that target the active GTP-bound state RAS(ON) for both mutant and wild-type RAS have shown promising results for KRASG12V-mutant cancers [71]. The first in class of these inhibitors, RMC-6232, forms a tricomplex with RAS(ON) and an abundant intracellular chaperon protein cyclophilin A, sterically inhibiting RAS binding to its effectors [72,73]. RMC-6232 is being assessed in a phase I clinical trial for KRASG12-mutant tumors (Table 1) and appears effective against KRAS position 12 (G12X) mutants, including G12D, G12V and G12R, inducing durable suppression of the RAS pathway activation in preclinical cellular in vitro and in vivo xenograft PDAC models [66,73].

### 3.5. Toxicity Challenges

As researchers explore the dynamic space of targeted therapy combinations in pancreatic cancer, the optimism of the recent advancements is tempered by the potential for overlapping toxicities in regimens incorporating two inhibitors targeting within the RAF/MEK/ERK pathway or combined with other targets, like in the case of dual inhibition with afatinib and trametinib [74]. Targeting the upstream regulators of the RAF/MEK/ERK pathway, SHP2 and SOS1, holds promise but at the same time raises concerns about unanticipated on-target toxicities, as evidenced by clear dose-associated cytopenias [75,76]. The clinical trial combining the KRASG12C inhibitor, sotorasib, with the anti-PD1 and anti-PD-L1 monoclonal antibodies pembrolizumab and atezolizumab, respectively, revealed increased liver toxicities [77]. The mechanisms driving these toxicities remain elusive, prompting hypotheses ranging from enhanced immune-mediated effects triggered by targeted therapies to potential off-target covalent protein–drug conjugates causing liver damage, exacerbated by systemic immune activation. Interestingly, Genentech’s GDC-6036, a KRASG12C inhibitor administered at lower doses, has shown reduced liver toxicities in phase I testing, suggesting that dosage adjustments may play a crucial role in mitigating adverse effects [78]. Preclinical studies of the RMC-6236 tricomplex have shown success in inhibiting active RAS(ON), including cases of acquired resistance by KRASG12C inhibitors, but the ubiquitous nature of cyclophilin A introduces uncertainties about the therapeutic window and potential off-target activity [76,79]. Amidst the hope for breakthroughs in treating pancreatic cancer, the toxicity challenges underscore the critical need for meticulous exploration of treatment schedules, adjustments and a deep understanding of the intricate interplay within the complex signaling pathways [76].

## 4. Discussion—Future Perspectives

The presence of KRAS mutations in pancreatic cancer has significant prognostic implications, influencing both overall survival (OS) and treatment response. Patients with KRAS-mutated PDAC generally exhibit a poorer prognosis [80]. Furthermore, recent findings indicate distinct survival outcomes related to specific KRAS mutations. For instance, patients with KRASG12D-mutated PDAC demonstrated a significantly shorter median overall survival compared to those with KRASG12R mutations, indicating a prognostic value for KRASG12D mutation [81,82]. Notably, the type of KRAS mutation may also impact the response to first-line chemotherapy. Thus, FOLFIRINOX showed improved survival in patients with KRASG12D and KRASG12V mutations, while the patients with KRASG12C-mutated tumors exhibited longer overall survival when treated with gemcitabine plus nab-paclitaxel [83]. Additionally, the variant allele frequency (VAF) and allelic imbalance of KRAS further contribute to prognosis. Higher KRAS VAF is associated with shorter survival, and allelic imbalance, leading to increased mutant KRAS dosage, correlates with a more aggressive clinical behavior [84,85]. Beyond KRAS, BRAF mutational status seems to have prognostic value. In a case report, two PDAC patients who had not responded to initial systemic chemotherapy, after identification of BRAFV600E mutation through next generation sequencing, were treated with combined dabrafenib and trametinib and sustained a favorable response [33]. These findings underscore the importance of molecular profiling, specifically KRAS mutation characterization, in guiding prognosis and tailoring therapeutic strategies for pancreatic cancer patients.

The omnipresent *KRAS* mutation in PDAC and the progress in drug development of small-molecule inhibitors led the early therapeutic efforts targeting the main components downstream of the RAF/MEK/ERK pathway. Although preclinical studies demonstrated promising findings, the clinical attempts were unsuccessful due to low efficacy and dose-limiting toxicities [86]. However, when a precision medicine approach was followed the paradigm was shifted. Case reports indicate benefits when the BRAF mutational status was confirmed, in a wild-type KRAS context, before therapeutic intervention [29,30,31,32,33,34]. Pharmacological targeting of the canonical components of RAF/MEK/ERK signaling in RAS-dependent tumors is often limited by the development of adaptive resistance, which is usually mediated by feedback activation of RTK signaling, resulting in reactivation of the RAF/MEK/ERK pathway activity [53]. Thus, the strategy of targeting additional effectors downstream of RTKs and upstream of RAS, such as SHP2 and SOS1, is attractive. Therapies that directly target the mutated components of the pathway such as KRAS or BRAF could be combined with inhibitors against upstream regulators such as SHP2 and SOS1 and downstream pathway components such as MEK or ERK for a sustained inhibition (Table 1). The concept of dual pathway inhibition has been successfully tested in the case of *BRAFV600E*-mutated melanoma, where vertical double BRAF/MEK inhibition has gained FDA approval. Furthermore, in the context of *BRAFV600E*-mutant tumors there is evidence for effectiveness of a triple inhibitory strategy within the RAF/MEK/ERK pathway [87]. A recent clinical trial, based on promising preclinical data, tested the combination of avutometinib, a first-in-class RAF/MEK clamp and a compound designed to inhibit MEK and block RAF-mediated phosphorylation of MEK in combination with the focal adhesion kinase (FAK) inhibitor defactinib (Table 1, Figure 1). The recent approval of the *KRASG12C* inhibitor sotorasib for *KRASG12C*-mutated non-small cell lung cancer (NSCLC) allowed the enrolment of low-frequency *KRASG12C*-PDAC cases in clinical trials for evaluation (Table 1). Currently, direct inhibition of mutant RAS through allele-specific inhibitors provides a therapeutic opportunity. Interestingly, inhibition of KRASG12D, using MRTX1133, in immunocompetent PDAC models resulted in tumor suppression by increasing tumor-associated macrophages (TAMs) and tumor-infiltrating cytotoxic T cells [88]. This argues that KRASG12D inhibition has a potential immunomodulatory function, which may be beneficial especially for patients with pancreatic cancer, an immunologically “cold” malignancy.

## 5. Conclusions

Despite the progress in drug discovery, there is an additional need to develop novel, more potent and broader RAF/MEK/ERK pathway inhibitors, including KRAS-mutant inhibitors, for improved tailored targeted therapy [89]. Developing effective and mechanism-based combination therapy regimens is essential to maximizing the efficacy of RAF/MEK/ERK pathway inhibition, which holds great promise for pancreatic cancer control.

## Figures and Tables

**Table 1:** RAF/MEK/ERK pathway inhibitors currently in clinical evaluation for pancreatic cancer. EGFR, epidermal growth factor receptor; ERBB2/HER2, receptor tyrosine-protein kinase erbB 2; RAF, rapidly accelerated fibrosarcoma; SHP2, Src homology 2 domain-containing phosphatase 2; SOS1, Son of sevenless homolog 1; KRAS, Kirsten rat sarcoma viral oncogene homolog; RAS, rat sarcoma viral oncogene homolog; RAF, rapidly accelerated fibrosarcoma; MEK, mitogen-activated protein kinase kinase; ERK, extracellular signal-regulated kinase; CDK4/6, cyclin-dependent kinase 4/6; HDAC, histone deacetylase; PARP, poly-adenosine diphosphate (ADP) ribose polymerase; eIF4A, eukaryotic translation initiation factor 4A; ULK1/2, unc-51-like autophagy-activating kinases 1 and 2; NLRP3, Nod-like receptor protein 3; PD-1, programmed cell death protein 1; PD-L1, programmed death ligand 1; VEGF-A, vascular endothelial growth factor A; PI3Kα, phosphoinositide 3-kinase α; FAK, focal adhesion kinase; JAK1/2, Janus kinase 1/2.

Drug(s)	Target(s)	Second Drug(s)	Second Target(s)	Phase	Clinical Study Code
Neratinib	EGFR, ERBB2/HER2	Divalproex sodium (Valproate)	HDAC	I/II	NCT03919292
Vemurafenib	BRAFV600E/K	Sorafenib	RAF	II	NCT05068752
Lilirafenib	BRAF	Mirdametinib	MEK	I	NCT03905148
Tovorafenib	RAF	Pimasertib	MEK	I/II	NCT04985604
Avutometinib	MEK, RAF	Defactinib	FAK	I/II	NCT05669482
ABM-168	MEK			I	NCT05831995
Binimetinib	MEK	Hydroxychloroquine	Autophagy	I	NCT04132505
Binimetinib	MEK	Encorafenib	RAFV600E/K	II	NCT04390243
Binimetinib	MEK	Palbociclib	CDK4/6	II	NCT05554367
Trametinib	MEK	Hydroxychloroquine	Autophagy	I	NCT03825289
Trametinib	MEK	Ruxolitinib	JAK1/JAK2	I	NCT04303403
Cobimetinib	MEK	Calaspargase pegol-mnkl (Asparlas)	Asparagine	I	NCT05034627
IMM-1-104	MEK			I/II	NCT05585320
Temuterkib	ERK	RMC-4630	SHP2	I	NCT04916236
Temuterkib	ERK	Hydroxychloroquine sulfate	Autophagy	II	NCT04386057
Ulixertinib	ERK	Palbociclib	CDK4/6	I	NCT03454035
ERAS-007	ERK	Encorafenib Palbociclib Cetuximab *	BRAFV600E/K CDK4/6 EGFR	I/II	NCT05039177
BI-1701963	SOS1	Adagrasib	KRASG12C	I	NCT04975256
BI-1701963	SOS1	Trametinib	MEK	I	NCT04111458
HBI-2376	SHP2			I	NCT05163028
JAB-3068	SHP2			I/II	NCT03565003
JAB-3312	SHP2			I	NCT04045496
JAB-3312	SHP2	Binimetinib Pembrolizumab * Sotorasib Osimertinib	MEK PD-1 KRASG12C EGFR	I/II	NCT04720976
BBP-398	SHP2	Sotorasib	KRASG12C	I	NCT05480865
RMC-6291	KRASG12C			I	NCT05462717
RMC-6291	KRASG12C	RMC-6236	RAS (pan-mutant and wild-type)	I	NCT06128551
HBI-2438	KRASG12C			I	NCT05485974
LY3537982	KRASG12C			I	NCT04956640
JAB-21822	KRASG12C			II	NCT06008288
JAB-21822	KRASG12C	Cetuximab *	EGFR	I/II	NCT05002270
JAB-21822	KRASG12C	JAB-3312	SHP2	I/II	NCT05288205
Adagrasib	KRASG12C			I	NCT05634525
Adagrasib	KRASG12C	TNO155	SHP2	I/II	NCT04330664
Adagrasib	KRASG12C	Afatinib Cetuximab * Pembrolizumab *	EGFR/HER2 EGFR PD-1	I	NCT03785249
Adagrasib	KRASG12C	Olaparib	PARP	I	NCT06130254
Adagrasib	KRASG12C	BMS-986466 ^†^ −/+ cetuximab *	NLRP3 EGFR	I/II	NCT06024174
Adagrasib	KRASG12C	MRTX0902	SOS1	I/II	NCT05578092
BPI-421286	KRASG12C			I	NCT05315180
BI-1823911	KRASG12C	BI-1701963	SOS1	I	NCT04973163
Divarasib	KRASG12C	Atezolizumab * Cetuximab * Bevacizumab * Erlotinib GDC-1971 Inavolisib	PD-L1 EGFR VEGFA EGFR SHP2 PI3Kα	I	NCT04449874
Garsorasib	KRASG12C			I	NCT04585035
JNJ-74699157	KRASG12C			I	NCT04006301
JDQ443	KRASG12C	TNO155 Tislelizumab *	SHP2 PD-1	I/II	NCT04699188
MK-1084	KRASG12C	Pembrolizumab *	PD-1	I	NCT05067283
Sotorasib	KRASG12C			I/II	NCT03600883
Sotorasib ^§^	KRASG12C			II	NCT04185883
Sotorasib	KRASG12C			I	NCT04380753
Sotorasib	KRASG12C	Panitumumab *	EGFR	II	NCT05638295
Sotorasib	KRASG12C	Panitumumab*	EGFR	II	NCT05993455
Sotorasib	KRASG12C	DCC-3116 ^†^	ULK1/2	I/II	NCT04892017
Sotorasib	KRASG12C	Zotatifin ^†^	eIF4A	I/II	NCT04092673
MRTX1133	KRASG12D			I/II	NCT05737706
RMC-9805	KRASG12D			I	NCT06040541
HRS-4642	KRASG12D			I	NCT05533463
RMC-6236	RAS (pan-mutant and wild-type)			I	NCT05379985
RSC-1255	RAS (pan-mutant and wild-type)			I	NCT04678648

* Monoclonal antibody; ^§^ as monotherapy or in combination with various anti-cancer agents; ^†^ main test drug of the trial.

## Data Availability

Data are contained within the article.
